# Supplementary material for: Zebrafish macrophages convert physical wound signals into rapid vascular permeabilization
Source: Nat Commun. 2026 Feb 6;17:1807. doi: 10.1038/s41467-026-68520-2 (PMC12916771; doi:10.1038/s41467-026-68520-2)
Supplement: Supplementary file 1 — Supplementary Information [file 41467_2026_68520_MOESM1_ESM.pdf]

Zebrafish macrophages convert physical wound signals into rapid vascular permeabilization

Zaza Gelashvili[1,2], Zhouyang Shen[1,2,3], Yanan Ma[1], Mark Jelcic[1,4], Philipp Niethammer[1*]

[1]Cell Biology Program, Memorial Sloan Kettering Cancer Center, New York, NY, USA

[2]Louis V. Gerstner, Jr. Graduate School of Biomedical Sciences, Memorial Sloan Kettering Cancer Center, New York, NY, USA

[3]Bloomberg-Kimmel Institute for Cancer Immunotherapy, Department of Oncology, Johns Hopkins University School of Medicine, Baltimore, MD, USA,

[4]Fate Therapeutics, Inc., San Diego, CA, USA

*Corresponding author, niethamp@mskcc.org

Supplementary Fig. 1

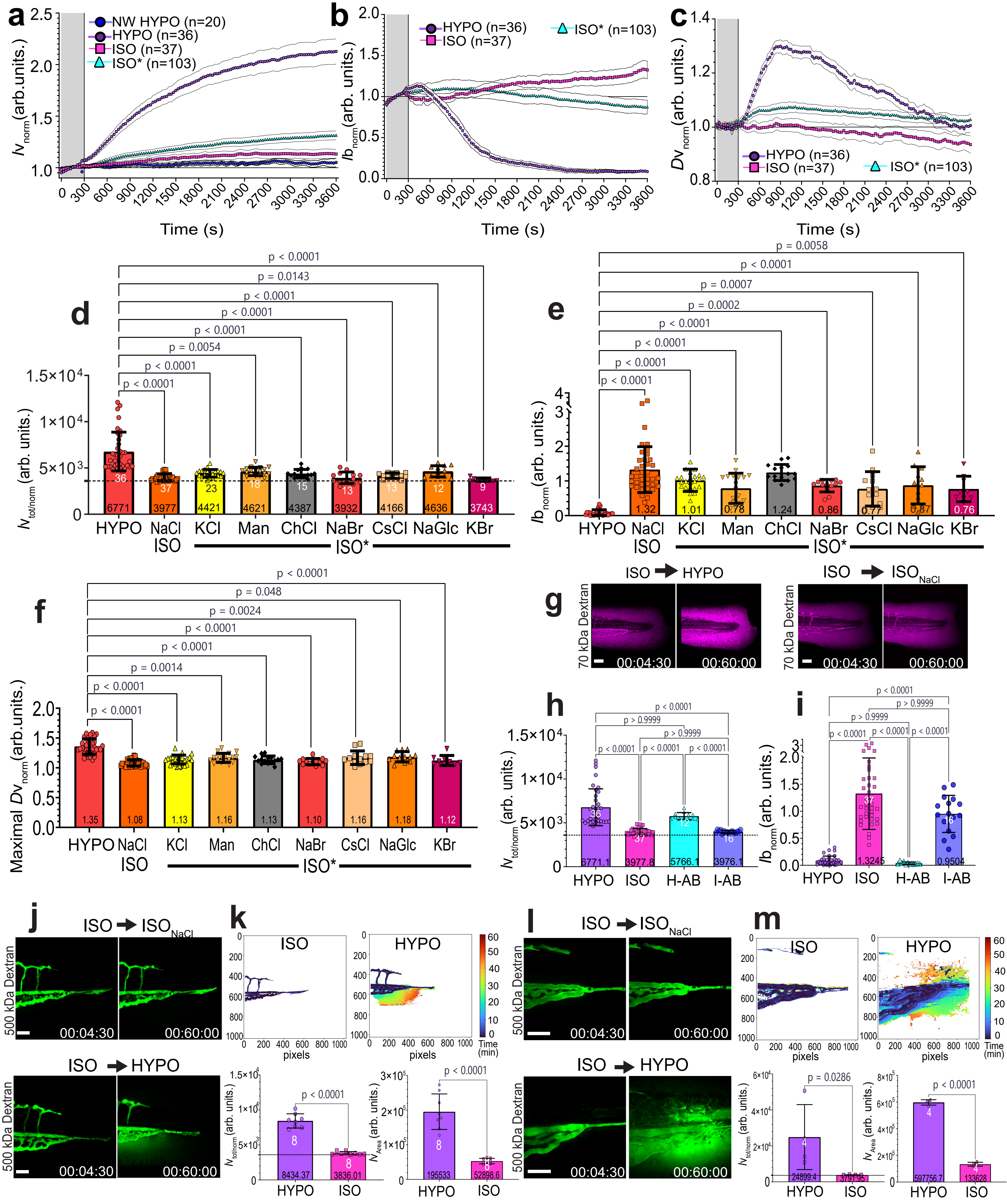

Supplementary Fig. 1. Hypoosmotic shock triggers vascular dilation and dextran leakage. Dynamics of normalized (**a**) vessel leakage ($I\mathrm{v}_{\mathrm{tot/norm}}$(t)), (**b**) wound leakage ($I\mathrm{b}_{\mathrm{norm}}$ (t)), and (**c**) vessel dilation ($D\mathrm{v}_{\mathrm{norm}}$(t)) at the indicated conditions. HYPO, regular E3. ISO, 280 mOsm NaCl or other osmolytes in E3. isotonic treatments KCl, Mannitol, ChCl, CsCl, NaGlc, KBr, NaBr. Lines, Average Error margins, 95% CI. (**d**) Integrated vessel leakage (t= 0-3600 s), (**e**) steady state wound leakage (t= 3600 s), and (**f**) maximal vessel dilation. Note, NW= Non-Wounded. For panels d-f, p values are indicated and determined using an unpaired, two-sided Kruskal-Wallis with Dunn's multiple comparison test. (**g**) Confocal maximal intensity projection (MIPs) of wounded 3dpf AB larvae before (t= 270 s) and after (t= 3600 s) switch of bathing solutions. Magenta, pseudo-coloured 70 kDa dextran fluorescence. (**h**) Quantification of dextran leakage ($I\mathrm{v}_{\mathrm{tot/norm}}$(t)) or (**i**) normalized dextran wound leakage ($I\mathrm{b}_{\mathrm{norm}}$). P values are indicated and determined using unpaired, two-sided Kruskal-Wallis test with Dunn's multiple comparison test (HYPO, n= 36 larvae; ISO, n= 37 larvae; HYPO-AB, n= 12 larvae; ISO-AB, n= 16). (**j**) Confocal MIPs of wounded casper zebrafish larvae with 500kDa Dextran injection, before and after shift of the indicated bathing solutions. Green, 500kDa Dextran Fluorescence. (**k**) Top panel, kymographs (t= 0-3600 s) of 500 kDa dextran leakage, track colour indicates frame using a linear rainbow (turbo) LUT spanning the full colour range from the first frame to the last frame (0–60 min). Bottom Left panel, quantification of normalized, integrated 500 kDa dextran leakage ($I\mathrm{v}_{\mathrm{tot/norm},}$ T= 0-3600 s). Right panel, kymograph dextran vessel area measurement in complete FOV. P values are indicated and calculated using unpaired, two-tailed Welch's t-tests, (HYPO, n= 8 larvae; ISO, n= 8 larvae). (**l**) Confocal MIP of wounded casper zebrafish larvae injected with 500kDa dextran and x63 objective. (**m**) Top panel, time-coloured kymographs using the same linear rainbow (turbo) LUT as in (k). Bottom left panel, quantification of normalized, dextran leakage ($I\mathrm{v}_{\mathrm{tot/norm},}$ t= 0-3600 s). Right panel, kymograph area analysis from x63 objective. Indicated P values are calculated by unpaired, two-tailed Mann–Whitney U test. (HYPO, n= 4 larvae; ISO, n= 4 larvae). Scale Bars, 50 μm. Dashed line is hypothetical no leakage baseline= 3600 (arb. units.). White numbers, animals. Bottom of bar graph numbers, mean of dataset. bar plot error bars, SD. Source data are provided in the Source data file.

Supplementary Fig. 2

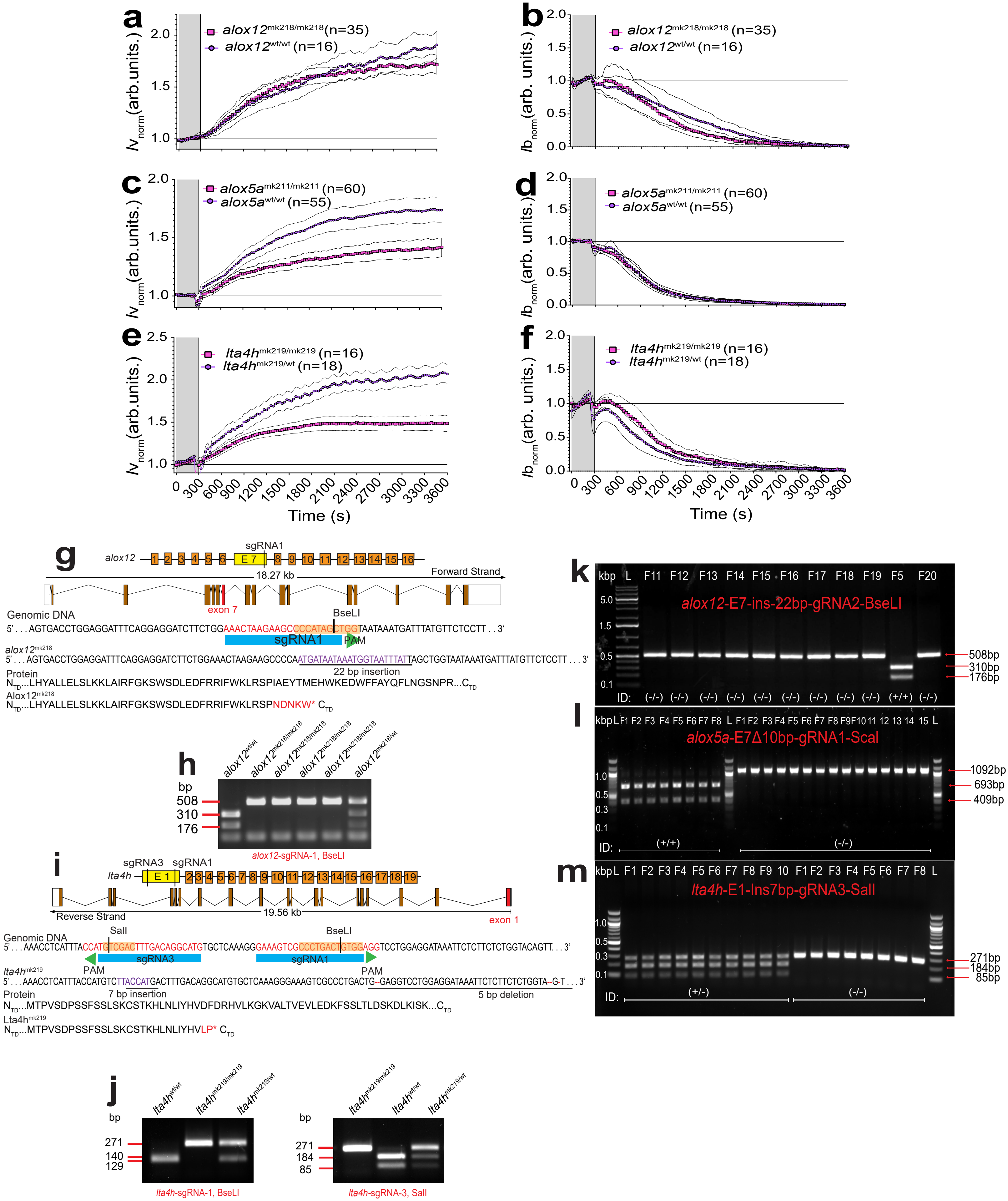

Supplementary Fig. 2. Alox5a–Lta4h signalling in osmotic vascular permeabilization. (**a-f**) Normalized (**a, c, e**) vessel- (Iv_{norm}(t)) and (**b, d, f**) wound- (Ib_{norm} (t)) leakage dynamics after ISO_{NaCl} → HYPO shifting at the indicated genetic conditions. Lines, Average. Error Margins, 95% CI. (**g**) Scheme illustrating the design guide RNAs targeting exon 7 of *alox12*. The Ensembl architecture of the exons (brown and red) and introns (black lines) in the *alox12* genomic region. The successful gene disruption is validated by BseLI (CCCATAG^CTGG) exon 7 restriction sites (yellow box) on genomic DNA, which is altered in *alox12*$^{mk218/mk218}$ mutants. (**h**) 486 bp polymerase chain reaction (PCR) product of the wildtype allele is cleaved into two fragments (310, 176bp) by BseLI. The mk218 allele carries a genomic 22bp insertion, preventing the targeted BseLI restriction enzyme digest in *alox12*$^{mk218/wt}$ (bp= 508, 310, 176) and *alox12*$^{mk218/mk218}$ (bp=508). (**i**) Scheme illustrating the design of two single guide RNAs targeting exon1 of *lta4h*. The Ensembl architecture of exons (brown and red) and introns (black lines) in the *lta4h* genomic region. Successful gene disruption is validated by BseLI (CCTGACT^GTGG) and SalI (G^TCGAC) exon1 restriction sites (yellow box) on genomic DNA, which is altered in *lta4h*$^{mk219/mk219}$ mutants. (**j**) The 269 bp PCR product of the wildtype allele is cleaved into two BseLI (140 and 129 bp) or SalI (184 and 85 bp) fragments. The mk219 allele carries a genomic 7bp insertion and 5bp deletion, preventing the targeted SalI or BseLI restriction enzyme digest in *lta4h*$^{mk219/wt}$ (bp= 271, 140 129; 271, 184 85) and *lta*$_4$*h*$^{mk219/mk219}$ (271bp). The F0 heterozygotes were identified by three distinct gel bands corresponding to PCR product enzyme digest of adult tailfins. (**k-m**) Mutations were validated for dextran injected larva after imaging. Representative ethidium bromide DNA with set PCR product restriction enzyme digest with (**k**) BseLI (bp=310, 176) for *alox12*$^{mk218/mk218}$ (bp=486) and *alox12*$^{wt/wt}$ (bp=310, 176), (**l**) ScaI (bp=693, 409) *alox5a*$^{mk211/mk211}$ (bp=1102) and *alox5a*$^{wt/wt}$ (bp=693, 409) (**m**) and SalI (bp=184, 85bp) *lta4h*$^{mk219/mk219}$ (bp=271) and *lta4h*$^{mk219/wt}$ (bp= 271, 184, 85), where each well corresponds to a single larva. n=number of animals. Exon-Intron schematics were adapted from Ensembl (Danio rerio, GRCz11). Source data are provided in the Source Data File.

Supplementary Fig. 3

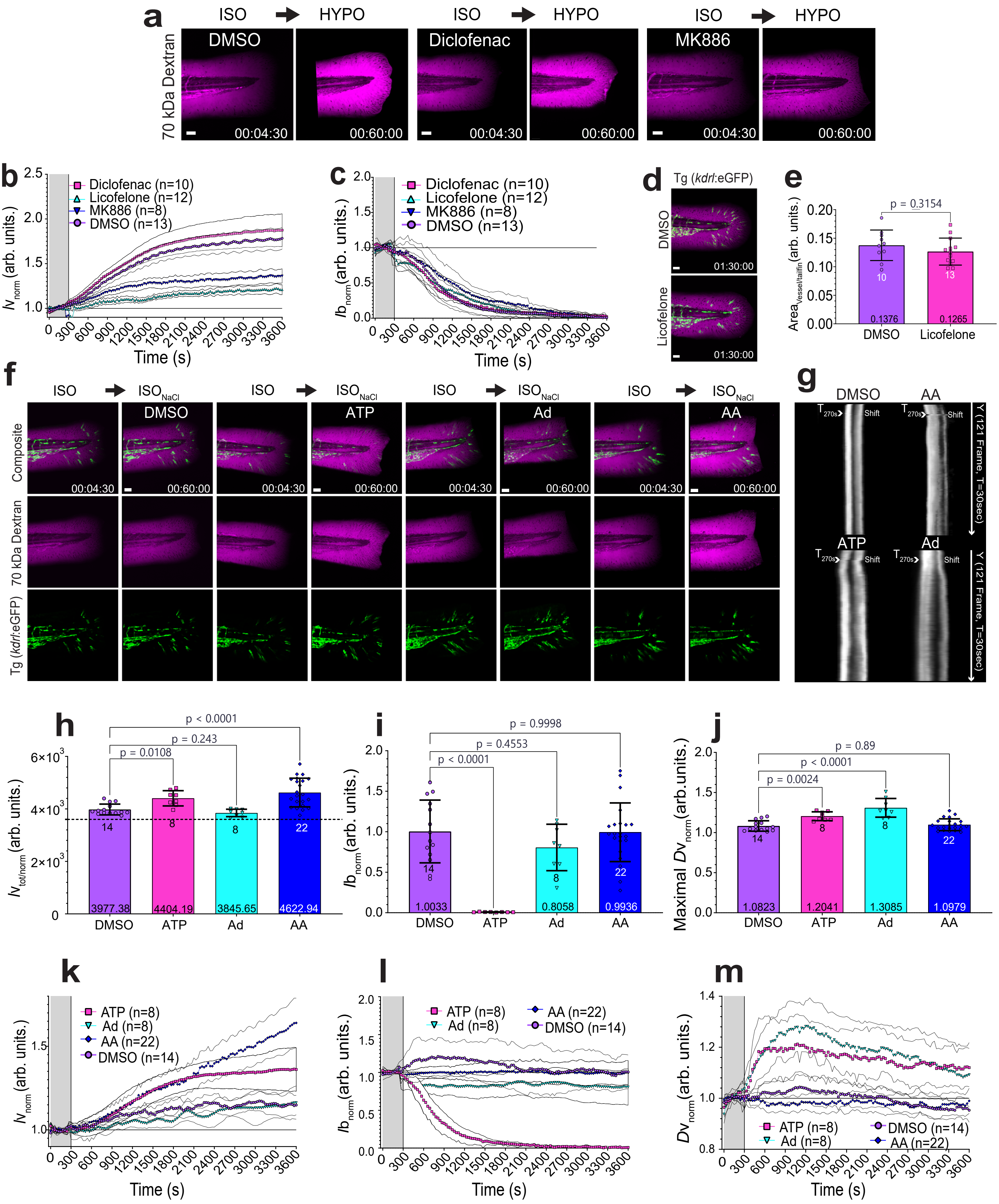

Supplementary Fig. 3. Pharmacological modulation of osmotic vascular permeabilization. (**a**) Representative confocal maximal intensity projection (MIPs) of pharmacologically pre-treated, wounded 3dpf zebrafish larvae before (t= 270s) and after (t= 3600s) switch of bathing solutions. Pharmacological antagonists. DMSO, vehicle. Diclofenac (130nM), licofelone (50µM) and MK886 (10µM). Magenta, pseudo-coloured 70 kDa dextran fluorescence. (**b**) Normalized vessel-(Iv_{norm}(t)) and (**c**) wound- (Ib_{norm} (t)) leakage dynamics after ISO_{NaCl} to HYPO shift at the indicated pharmacologic conditions. Lines, Average. Error Margin, 95% CI. (**d**) Representative confocal MIPs of pharmacologically pre-treated, non-wounded 3dpf (Tg(*kdrl*:eGFP)) larvae and imaged at 90 minutes post-treatment. Green, *kdrl:*eGFP fluorescence. Magenta, pseudo-coloured 70 kDa dextran fluorescence. (**e**) Quantification of vessel·tailfin^{-1} area ratio as function of vehicle (DMSO) or licofelone treatment. P values are indicated, and statistical significance was assessed using unpaired, two-tailed Welch's t-test, (DMSO, n= 10 larvae; licofelone, n= 13 larvae). (**f**) Representative Confocal MIPs of pharmacological agonists in wounded 3dpf (Tg(*kdrl*:eGFP)) larvae before (t= 270s) and after (t= 3600s) switch of bathing solutions. (**g**) Representative Kymographs for vessel analysis with indicated agonists. DMSO, vehicle. ATP, adenosine triphosphate (5 mM). Ad, adenosine (5 mM). AA, arachidonic acid (5 µM). Plots of normalized, (**h**) integrated vessel leakage (t= 0-3600 s), (**i**) steady state wound leakage (t= 3600 s), and (**j**) maximal vessel dilation upon treatment of wounded zebrafish larvae bathed in ISO_{NaCl} with the indicated agonists. For panels, h-j, p value is indicated and determined using unpaired, two-sided Welch's one-way ANOVA with Dunnett's T3 multiple comparison test, (DMSO, n= 14 larvae; ATP, n= 8 larvae; Ad, n = 8 larvae; AA, n= 22 larvae). Normalized vessel leakage (Iv_{norm}(t)), (**k, l, m**) wound leakage (Ib_{norm} (t)), and vessel dilation dynamics after ISO_{NaCl} to ISO shifting with the indicated agonists. Numbers below the error bars, animals. Numbers at the bottom of bar graph, mean of dataset. Bar plot error bars, SD. Scale Bars, 50 µm. Dashed line is hypothetical no leakage baseline= 3600 (arb. units.) Note, (h-j) and (k-m) refer to the same experiment. Source data are provided in the Source Data File.

Supplementary Fig. 4

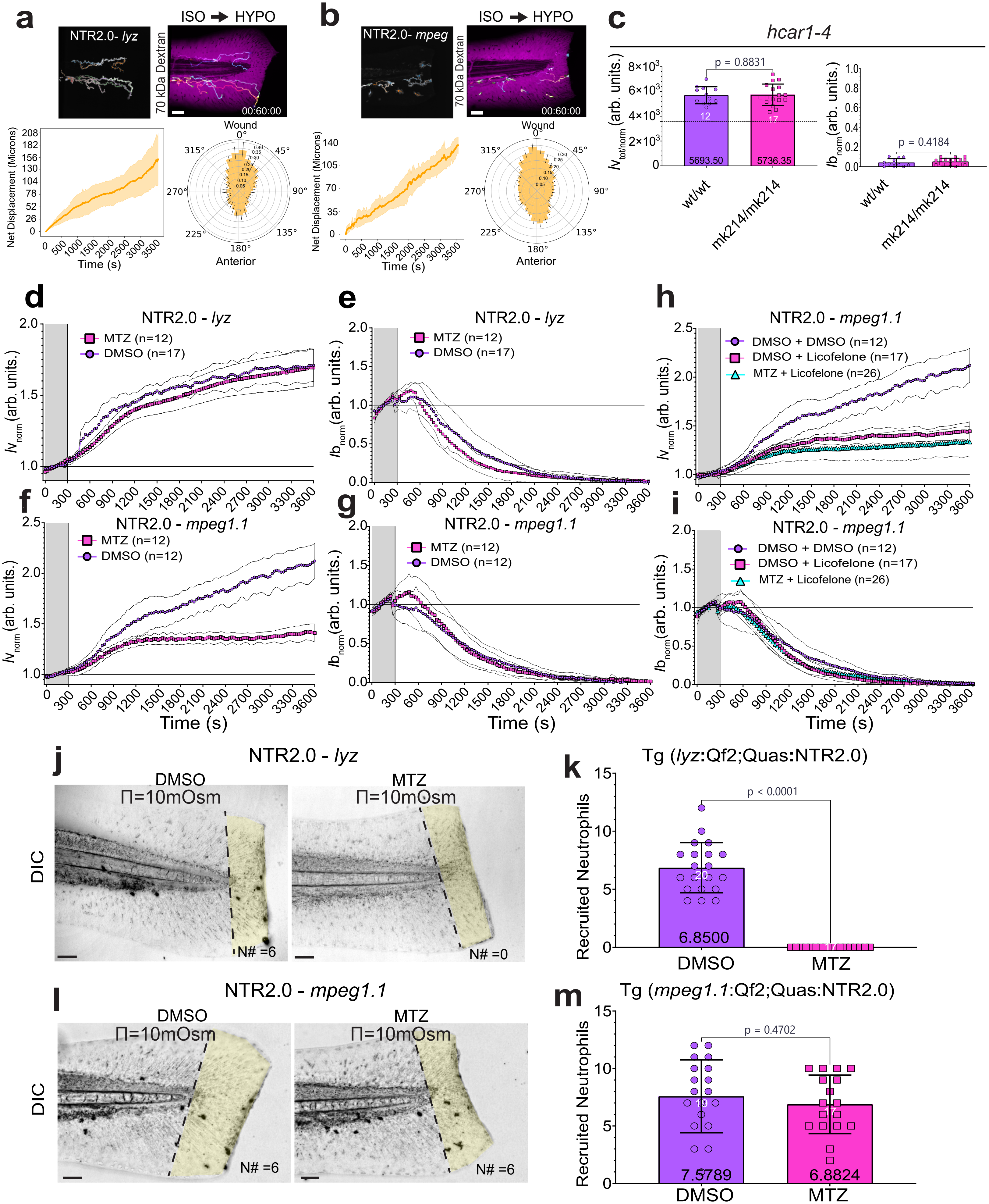

Supplementary Fig. 4. Chemogenetic depletion of neutrophils and macrophages in vascular permeabilization. (**a**)Top left, neutrophil kymograph overlayed with coloured nearest-neighbour tracks. Right panel, neutrophil tracks on 70kDa Dextran (magenta pseudocolor) overlay. Bottom left, time-series plot for neutrophil displacement. Bottom right, angular rose polarity plot, (A-P axis: 0 °= Wound, 180 °= Anterior). (**b**) Top left, representative kymograph overlayed with macrophage tracks. Bottom left, macrophage displacement as function of time and polarity plot. Lines, Average. Error margin, 95% CI. Right, macrophage angular rose plot (A-P axis: 0 °= Wound, 180 °= Anterior). Polarity plot line, average and error bars, 95% CI. Error margin, 95 % CI. Note data was derived from Fig. 3b, d. (**c**) Bar plot quantification of normalized 70kDa dextran vessel- (Iv_{norm}(t)) and wound- leakage in 3dpf homozygous (*hcar1-4*$^{mk214/mk214}$) mutant and wildtype (*hcar1-4*$^{wt/wt}$) hcar1-4 animals. P values are indicated and determined using unpaired, two-tailed Welch's t-test, (wt/wt, n= 12 larvae; mk214/mk214, n= 17 larvae). Dashed line is leakage baseline= 3600 (arb. units). Normalized (**d**-**i**) vessel- (Iv_{norm}(t)) and wound- (Ib_{norm} (t)) leakage dynamics after ISO_{NaCl} to HYPO shift for NTR2.0-*lyz* (**d**,**e**), NTR2.0-*mpeg* (**f**, **g**, **h**, **i**) at indicated conditions. Lines, Average. Error Margin, 95% CI. (**j**) Left panel, representative image of Sudan black staining shown in control (DMSO) and neutrophil depleted (MTZ) tailfins of 4dpf Tg(*lyz*:Qf2;Quas:NTR2.0) larvae at 90 min post-injury. Neutrophils are stained with Sudan Black. N#, number of recruited neutrophils in the tailfin region (highlighted in yellow). (**k**) Bar plot quantification of neutrophils in depletion and Sudan Black staining. P value is indicated and determined using unpaired, two-tailed Mann–Whitney U test, (DMSO, n= 20 larvae; MTZ, n= 17 larvae). (**l**) Representative macrophage depleted (MTZ) and vehicle (DMSO) tail fin images of 4dpf (Tg(*mpeg1.1*: Qf2;Quas:NTR2.0)) larvae at 90 min post-injury. Neutrophils, stained with Sudan Black. N#, number of recruited neutrophils in the tailfin region (highlighted in yellow). (**m**) Bar plot quantification of neutrophils in macrophage depletion and Sudan Black staining. P value is indicated and determined using unpaired, two-tailed Welch's t-test, (DMSO, n= 19 larvae; MTZ, n= 17 larvae). Scale bars, 50 μm. White numbers, animals. Black numbers, mean of dataset. Source data are provided in the Source Data File.

Supplementary Fig. 5

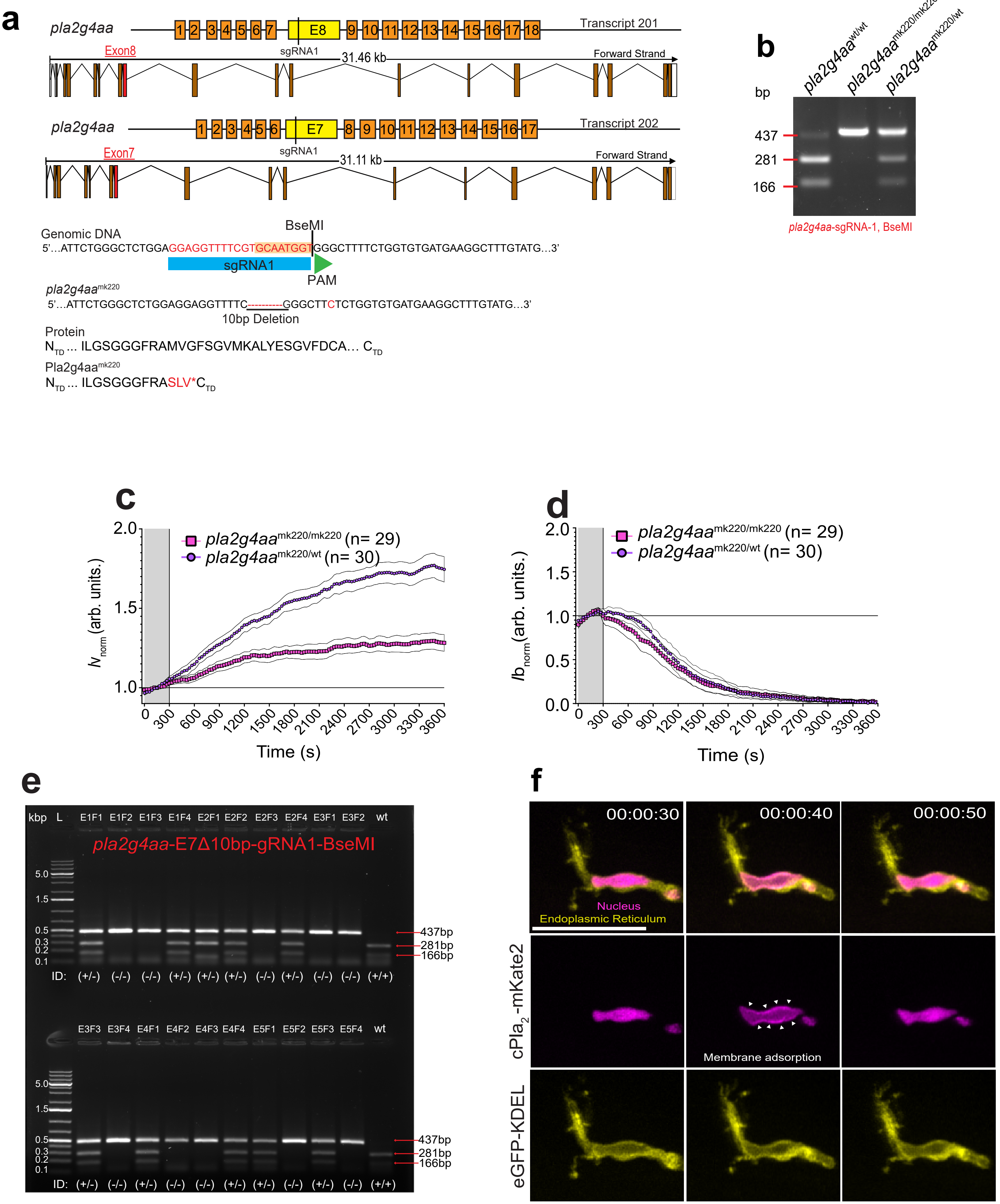

Supplementary Fig. 5. $cPLA_2$ couples osmotic stress to vascular permeabilization. (**a**) Scheme illustrating the design of a single guide RNA targeting exon8 (transcript 201), exon7 (transcript 202) of *pla2g4aa* mutant zebrafish. bottom the Ensembl architecture of exons (brown and red) and introns (black lines) in the zebrafish *pla2g4aa* genomic sequence. Successful gene disruption is validated with BseMI (GCAATGGT^) restriction palindrome sites (yellow box) on genomic DNA, which is altered in *pla2g4aa*$^{mk220/mk220}$ mutants. (**b**) Representative gel genotyping of CRISPR/Cas9 induced *pla2g4aa* mutations by BseMI restriction digest. The 447 bp PCR product of wildtype allele is cleaved by BseMI into two smaller (281 and 166 bp) fragments. The mk220 allele carries 10bp deletion, preventing the targeted BseMI restriction enzyme digest in *pla2g4aa*$^{mk220/mk220}$(437bp) and *pla2g4aa*$^{mk220/wt}$ (437, 281 and 166bp). Normalized (**c**) vessel- ($I\text{v}_{norm}$(t)) and (**d**) wound- ($I\text{b}_{norm}$ (t)) leakage dynamics after ISO_{NaCl} to HYPO shifting at the indicated genetic conditions. Lines, Average. Error Margin, 95% CI. n= number of larvae (**e**) Representative genotyping for *pla2g4aa*$^{mk220/wt}$ and *pla2g4aa*$^{mk220/mk220}$ larvae after image acquisition. Red arrows, restriction enzyme cleavage patterns. (**f**) Representative, pseudo-coloured $cPla_2$-mKate2 (magenta) and endoplasmic reticulum KDEL-eGFP (yellow) fluorescence in a perivascular macrophage before (00:00:30) and after (00:00:40 & 00:00:50) laser injury in HYPO. The arrows point to nuclear membrane adsorption in $cPla_2$ channel. Timestamp, hh:mm:ss. Scale bar, 25 µm. Exon-Intron schematics were adapted from Ensembl (Danio rerio, GRCz11). Source data are provided as a Source Data file.

Supplementary Fig. 6

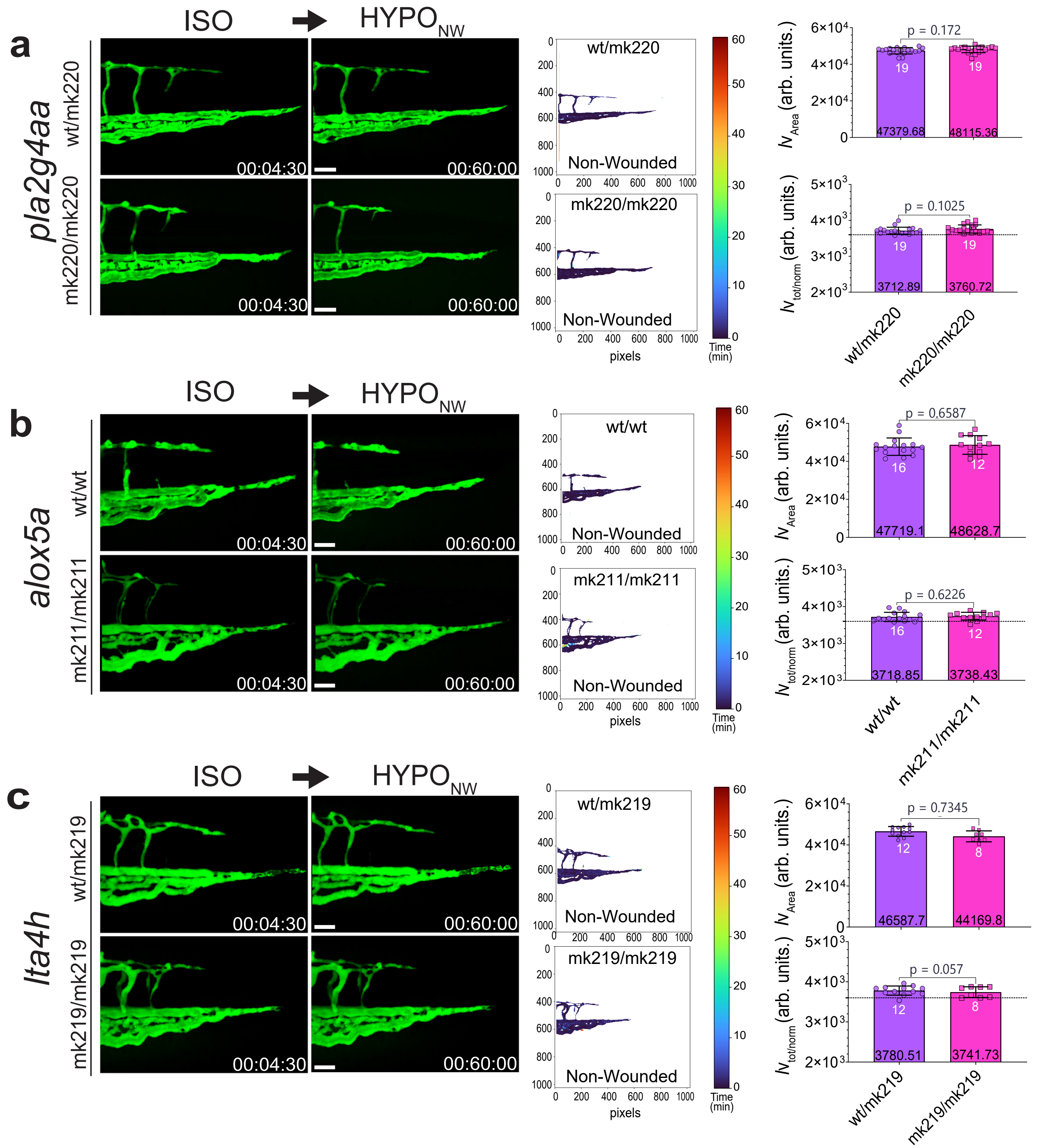

Supplementary Fig. 6. 500 kDa dextran and vascular morphology. (**a**) Left panel, cropped confocal Maximum Intensity Projection (MIPs) of NW (Non-Wounded) *pla2g4aa*$^{mk220/wt}$ heterozygous and mutant NW *pla2g4aa*$^{mk220/mk220}$ 3dpf animals before (t= 270s) and after (t= 3600s) switch of bath solutions. Mid panel, kymographs (t= 0-3600 s) of 500 kDa dextran leakage in NW *pla2g4aa*$^{mk220/wt}$ heterozygous and mutant NW *pla2g4aa*$^{mk220/mk220}$ animals before and after switch of indicated bathing solution from ISO_{NaCl} into HYPO. Right panels, top bar graph is quantification of vessel area from kymograph analysis. Bottom right bar plot, quantification of normalized, integrated (between, t= 0-3600 s) vessel leakage. P values are indicated and determined using unpaired, two-tailed Mann-Whitney U test (wt/mk220, n= 19 larvae; mk220/mk220*,* n= 19 larvae). (**b**) Left panel, 500 kDa dextran representative shown as cropped confocal MIPs of NW *alox5a*$^{wt/wt}$ (WT) and *alox5a*$^{mk211/mk211}$ mutant 3dpf animals before (t= 270s) and after (t= 3600s) switch of indicated bathing solution. Middle panel, full field of view (FOV) confocal MIPs kymographs (t= 0-3600 s). Top right bar graph, quantification of vessel area from kymograph analysis, bottom bar graph is quantification of normalized, integrated (between, t= 0-3600 s) vessel dextran leakage. P values are indicated and determined using unpaired, two-tailed Welch's t-tests, (wt/wt, n= 16 larvae; mk211/mk211, n= 12 larvae). (**c**) Left panel, cropped MIPs from 500kDa dextran and NW *lta4h*$^{wt/mk219}$ heterozygous and mutant *lta4h*$^{mk219/mk219}$ 3dpf animals before (t= 270s) and after (t= 3600s) switch of bath solutions. Middle panel, kymographs (t= 0-3600s) of 500 kDa dextran leakage from representative NW *lta4h*$^{wt/mk219}$ heterozygous and mutant *lta4h*$^{mk219/mk219}$ animals before and after switch of indicated bathing solution. Top right bar graph, vessel area from kymograph analysis. Bottom bar graph, normalized, integrated (between, t= 0-3600 s) vessel dextran leakage. P values are indicated and determined using unpaired, two-tailed Mann-Whitney U test (intensity) or Welch's t-test (area), (wt/mk219, n= 12 larvae; mk219/mk219, n= 8 larvae). Green, 500kDa dextran fluorescence. Timestamp, hh:mm:ss. Scale bars, 50 µm. Dashed line is hypothetical no leakage baseline= 3600 (arb. units). White numbers, number of animals. Bottom of bar graph numbers, mean of dataset. Source data are provided as Source Data file.

Supplementary Fig. 7

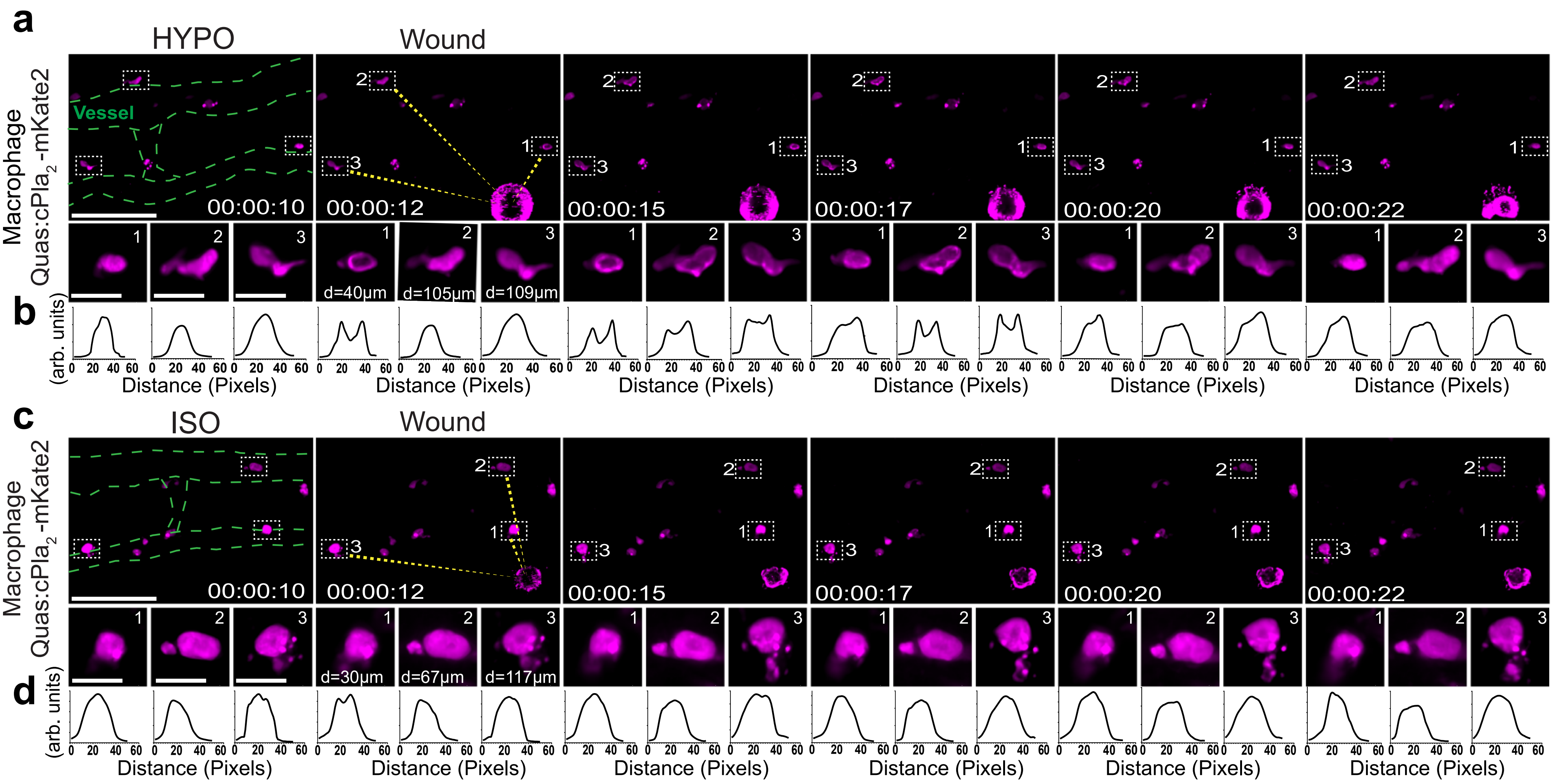

Supplementary Fig. 7. Time-resolved nuclear mechanotransduction *in vivo*. Representative two-photon montage of live 3dpf Tg(*mpeg1.1*:Qf2;Quas:cPla$_2$-mKate2) larvae imaged with rapid temporal resonant scanning (t-step= 2.5 s, 6 fps) upon laser injury at t= 12 s in (**a-b**) HYPO and (**c-d**) ISO$_{NaCl}$ E3 bathing solution. Magenta, pseudo-coloured for cPla$_2$-mKate2. Images are representative of three independent wounding experiments (HYPO, n=3 larvae; ISO, n=3 larvae). Dashed green line, vessel outline. Timestamp, hh:mm:ss. Scale bars, 50 µm. Inset scale bars, 10 µm (**b, d**) Line profiles for cPla$_2$-mKate2 emission(arb. units.) over distance (Pixels) through the macrophages in the numbered ROIs. Source data are provided as Source Data file.

Supplementary Fig. 8

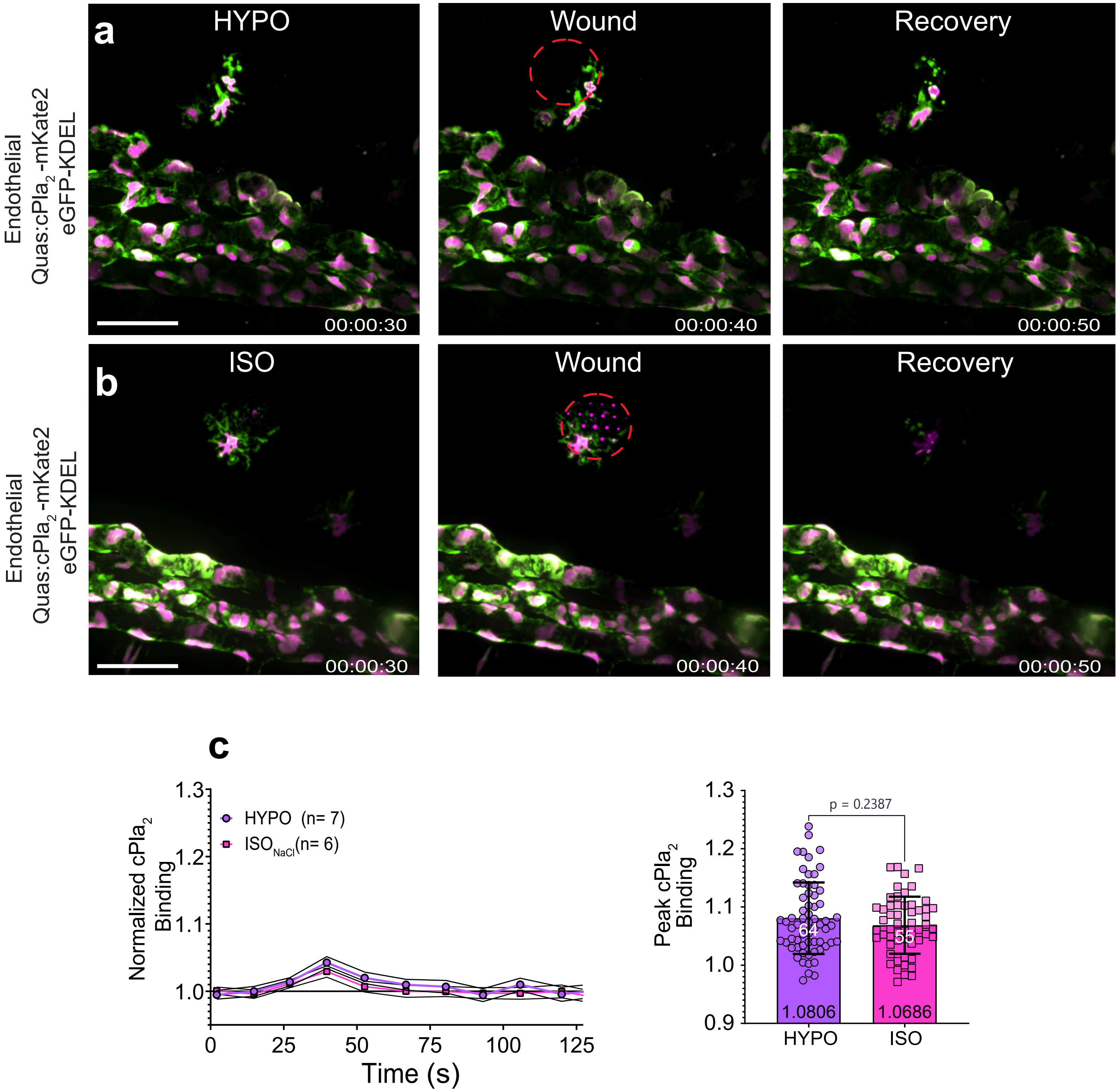

Supplementary Fig. 8. Endothelial cPla$_2$ activity in osmotic UV damage. Representative confocal maximum intensity projection (MIPs) of 3dpf Tg(*kdrl*:cPla$_2$-mKate2-p2a-eGFP-KDEL) larvae before and after laser injury in (**a**) HYPO (10 mOsm, upper panel) and (**b**) ISO (280 mOsm, lower panel) bathing solution. UV laser injury induced at t= 40 s UV-laser blast (dashed, red circle). Magenta pseudo-colour, *kdrl*: cPla$_2$-mKate2-eGFP-KDEL. Scale bars, 50 μm. Time stamp, hh:mm:ss. (**c**) Left panel, cPla$_2$-mKate2-INM binding dynamics. INM-binding of cPla$_2$-mKate2 is quantified as a ratio of perinuclear to nucleoplasmic fluorescence signal and normalized to its initial (t= 0 s) value within the endothelial cell layer. Right panel, comparison of peak translocation. N, number of analysed nuclei from 64 (n= 7 larvae) and 55 (n= 6 larvae) endothelial cells from HYPO and ISO$_{NaCl}$ laser wounds, respectively. P values are indicated and determined using an unpaired, two-tailed Welch's t-test (HYPO, n= 7 larvae, N=64 nuclei; ISO, n= 6 larvae, N= 55 nuclei). Error Margins, 95% CI, bar plot error bars, SD. White numbers, endothelial nuclei. Black numbers, mean of dataset. Source data are provided as Source Data file.

Supplementary Fig. 9

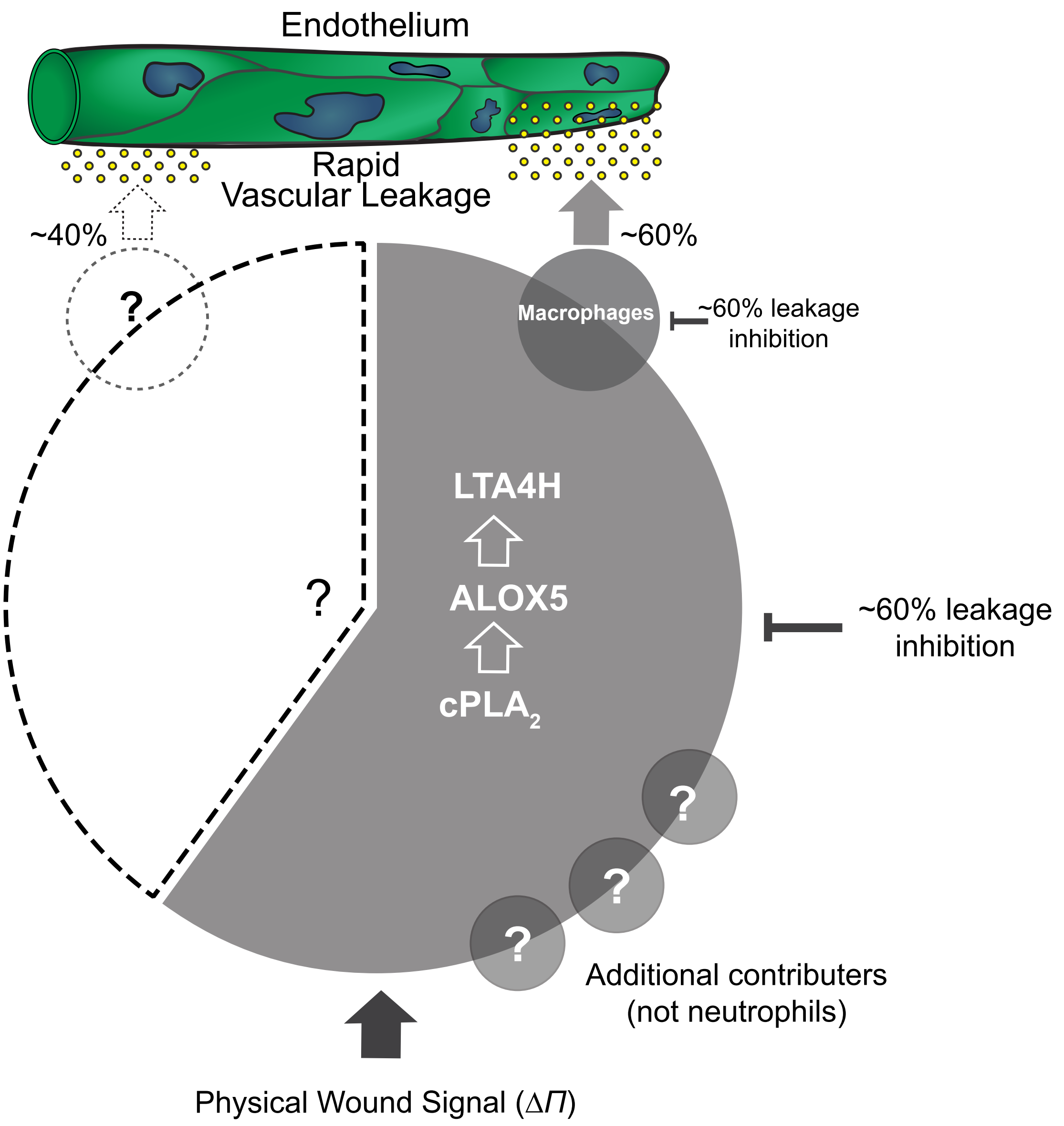

Supplementary Fig. 9 Schematic summary of osmotic vascular permeability. Cartoon scheme of working model. Rapid vascular leakage almost entirely depends on an osmotic signal ($\Delta\Pi$). Inhibition of the cPLA2-ALOX5-LTA4H pathway (grey shading) by genetic or pharmacologic perturbation reduces vascular leakage by ~60% (see pathway pie chart,). Without macrophages, leakage is also reduced by ~60% and becomes insensitive to ALOX5 perturbation. Thus, physically stimulated vessel permeabilization through the cPLA2-ALOX5-LTA4H pathway is mediated by macrophages. Our data neither indicate nor exclude contributions of other cell types and mechanotransduction pathways (question marks, and dotted lines).
