# Supplementary material for: Zebrafish macrophages convert physical wound signals into rapid vascular permeabilization
Source: Nat Commun. 2026 Feb 6;17:1807. doi: 10.1038/s41467-026-68520-2 (PMC12916771; doi:10.1038/s41467-026-68520-2)
Supplement: Supplementary file 2 — Description of Additional Supplementary Files [file 41467_2026_68520_MOESM2_ESM.pdf]

Description of Additional Supplementary Files

Supplementary Movie 1: Representative time lapse movie of a 2.5 dpf zebrafish Tg(*kdrl*:eGFP)-larva showing vessel dilation and vessel permeability upon shifting of bathing solutions from ISO to HYPO. Overexposure of dextran emission is to highlight the bleed out from the wound. Magenta, 70 kDa dextran. Green, vessels. Timestamp, hh:mm:ss. Scale bars, 50 µm.

Supplementary Movie 2: Representative time lapse movie of a dextran-injected and wounded 2.5 dpf Tg(*kdrl*:eGFP)-larva showing inhibition of vessel dilation and permeability upon shifting of bathing solutions from ISO to ISO_{NaCl}. Magenta, 70 kDa dextran. Green, vessels. Timestamp, hh:mm:ss. Scale bars, 50 µm.

Supplementary Movie 3: Representative time lapse movie of a dextran-injected and - wounded 2.5 dpf Tg(*kdrl*:eGFP)-larva showing mild inhibition of vessel dilation and permeability upon shifting of the bathing solution from ISO to ISO_{ChCl}. Magenta, 70 kDa dextran. Green, vessels. Timestamp, hh:mm:ss. Scale bars, 50 µm.

Supplementary Movie 4: Representative time lapse movie of 500kDa dextran injected and wounded 2.5 dpf casper zebrafish larvae, showing inhibition of vessel wound detection upon shift of bathing solutions (20x and 63x objective) from ISO to ISO_{NaCl}. Green, 500kDa Dextran. Timestamp, hh:mm:ss. Scale bars, 50 µm.

Supplementary Movie 5: Representative time lapse movie of 500kDa dextran injected and wounded 2.5 dpf casper zebrafish larvae, showing vessel wound response upon shift of bathing solutions (20x and 63x objective) from ISO to HYPO. Green, 500kDa Dextran. Timestamp, hh:mm:ss. Scale bars, 50 µm.

Supplementary Movie 6: Representative time lapse movie of a dextran-injected and -wounded 3 dpf Tg(*lyz*:NTR2.0) -larva showing that neutrophil depletion does not impact vessel- or wound-permeability under hypotonic conditions. Green, neutrophils. Magenta, 70 kDa dextran. Timestamp, hh:mm:ss. Scale bars, 50 mm.

Supplementary Movie 7: Representative time lapse movie of a dextran-injected and -wounded 3 dpf Tg(*mpeg1.1*:NTR2.0)-larva showing that absence of macrophages inhibits vessel leak-age without impacting wound permeability. Green, macrophage. Magenta, dextran 70 kDa. Timestamp, hh:mm:ss. Scale bars, 50 mm.

Supplementary Movie 8: Representative time lapse movie of $cPla_2$-mKate2 emission in macrophage nuclei under HYPO and ISO treatments, showing membrane binding after UV-laser wounding (at t= 00:00:40) and rapid recovery (at t= 00:00:50) in HYPO bathing condition. Timestamp, hh:mm:ss. Scale bars, 50 mm.

Supplementary Movie 9: Representative two-photon imaging of macrophage $cPla_2$-mKate2 emission with fast temporal acquisition in HYPO and ISO treated zebrafish larvae showing that rapid and reversible $cPla_2$-INM adsorption propagates in wave-like fashion from the site of laser injury (at t= 00:00:12). Timestamp, hh:mm:ss. Scale bars, 50 mm.

Supplementary Movie 10: Representative time lapse movie of a 2 dpf zebrafish larvae showing $cPla_2$-mKate2 emission in endothelial nuclei under HYPO and ISO treatments and UV-laser wounding (at t= 00:00:40). Timestamp, hh:mm:ss. Scale bars, 50 mm.
