# Supplementary material for: Zebrafish macrophages convert physical wound signals into rapid vascular permeabilization
Source: Nat Commun. 2026 Feb 6;17:1807. doi: 10.1038/s41467-026-68520-2 (PMC12916771; doi:10.1038/s41467-026-68520-2)
Supplement: Supplementary file 13 — Reporting Summary [file 41467_2026_68520_MOESM13_ESM.pdf]

nature portfolio

Corresponding author(s): Niethammer, Philipp

Last updated by author(s): Dec 3, 2025

Reporting Summary

Nature Portfolio wishes to improve the reproducibility of the work that we publish. This form provides structure for consistency and transparency in reporting. For further information on Nature Portfolio policies, see our Editorial Policies and the Editorial Policy Checklist.

Statistics

For all statistical analyses, confirm that the following items are present in the figure legend, table legend, main text, or Methods section.

n/a	Confirmed	
☐	☒	The exact sample size (n) for each experimental group/condition, given as a discrete number and unit of measurement
☐	☒	A statement on whether measurements were taken from distinct samples or whether the same sample was measured repeatedly
☐	☒	The statistical test(s) used AND whether they are one- or two-sided<br>*Only common tests should be described solely by name; describe more complex techniques in the Methods section.*
☒	☐	A description of all covariates tested
☐	☒	A description of any assumptions or corrections, such as tests of normality and adjustment for multiple comparisons
☐	☒	A full description of the statistical parameters including central tendency (e.g. means) or other basic estimates (e.g. regression coefficient) AND variation (e.g. standard deviation) or associated estimates of uncertainty (e.g. confidence intervals)
☐	☒	For null hypothesis testing, the test statistic (e.g. F, t, r) with confidence intervals, effect sizes, degrees of freedom and P value noted<br>*Give P values as exact values whenever suitable.*
☒	☐	For Bayesian analysis, information on the choice of priors and Markov chain Monte Carlo settings
☒	☐	For hierarchical and complex designs, identification of the appropriate level for tests and full reporting of outcomes
☒	☐	Estimates of effect sizes (e.g. Cohen's d, Pearson's r), indicating how they were calculated

Our web collection on statistics for biologists contains articles on many of the points above.

Software and code

Policy information about availability of computer code

Data collection	Zebrafish confocal imaging data was acquired using Nikon NIS-elements Software (NIS-Elements, 6.02.01; 5.12.0; 3.22.14). The data were collected under two different spinning-disc confocal microscopes housing CSU-W1 (NIS-Elements, 5.12.0) and CSU-X1 (NIS-Elements, 3.22.14) modules and equipped with a Photometrics back-illuminated Prime BSI Scientific CMOS (sCMOS) and Andor iXon3 897 thermoelectrically cooled EMCCD, respectively. The imaging plane (287 µm X 287 µm X 50-80 µm) was collected at 1.5µm z step size and repeated in no-delay intervals per position for up to 30 minutes, resulting with temporal step resolution of ~7-12 seconds per stack. The z-stacks were acquired at a resolution of 512 x 512 pixels (14-bit), corresponding to 0.561 µm/pixel calibration with a voxel size (0.56 X 0.56 X 1.5 µm) in x y and z, in the NIS imaging software (NIS Elements, 3.22.14). for sCMOS collection, XYZ-T image files were pre-processed by (2x2) binning and acquired as (1024 X 1024 per z-plane) with the following mage dimensions (665.6 x 665.6 x 100 µm) with 0.645 µm/pixel calibration in the NIS imaging software (NIS Elements, 5.12.0). The two-photon data was collected on external detectors (NDD 1&2) in resonant scanning mode(Pixel dwell time= 35ns, speed=8000 Hz) either in Nikon (NIS-Elements, 6.02.01) or in Leica Microsystems software (LAS X 4.8.1.29271). In Nikon (NIS-Elements, 6.02.01) MP imaging, the wounds were induced with mode-switch to galvanic scanning mode while on leica (LAS X 4.8.1.2971) the system was maintained in resonant mode. The z-stacks were acquired at a pixel resolution of 1024x626 (Nikon) or 1184x1184(Leica) (14-bit), corresponding to 0.144 µm/pixel calibration with a voxel size (0.14 X 0.14 X 1.5 µm) in x y and z in the NIS imaging software (NIS elements, 6.02.01). All obtained experimental files were written either as .nd2 or .lif files, all emission was collected as 14(16,384-levels) or 16(65,535-levels)-bit.
Data analysis	The experimental image .nd2 data were imported into Fiji (v1.54J, Just ImageJ Package) using bio-formats and PTBIOP plugins for import and drift correction. Zebrafish image stacks were maximum-intensity projected and analyzed in Fiji with plot z-axis profile function (https://github.com/zazadovv/Macrophage_Vessel.git). For endothelial signal and dilation, vertical line was drawn using line tool with fixed dimensions overlaying the registered endothelial fluorescence signal emission (length=50, width=30 pixel) and multi-kymograph function with default settings were used to generate kymograph images. The endothelial emission kymographs were binerized (Threshold method=Otsu,

Dark Background) to generate an 8-bit image and area quantified using makeline tool (width=30 pixels, length=121) and the diameter measured using the plot profile function. Whole field of view kymographs were also generated for vessels via Python (https://github.com/zazadovv/Macrophage_Vessel.git). The obtained vessel-related measurements and nuclear membrane binding quantifications were then subsequently analyzed in Python (Conda, v3.8.10 64-bit), and MATLAB (R2024a, V24.1.0.2537033, 64-bit) code and combined. source for these data analysis are publicly available at (https://github.com/joeshen123/Nuclear-Membrane-Binding-Analysis.git; https://github.com/zazadovv/Macrophage_Vessel.git). Representative images included in the manuscript were generated either in Fiji (v1.54J, Just ImageJ Package) or napari (v0.4.19). Plots generated in graphpad(v.10.2.3) or in matplotlib (v.3.5.2) and modified in adobe illustrator (2025.29.1.0). The 2D and 3D image analysis was performed with Anaconda distribution of Python (Python ≥ 3.8.10). Specifically, customized python scripts were developed using the Numpy (v.1.23.5), SciPy(v1.15.2) scikit-image (V0.19.1), matplotlib (v3.5),trackpy (v0.6.4), Allen Cell Structure Segmenter (v0.5.0) and napari (≥ v0.4.10) libraries. GPU acceleration (when available) was provided by CuPy (v12.0).

For manuscripts utilizing custom algorithms or software that are central to the research but not yet described in published literature, software must be made available to editors and reviewers. We strongly encourage code deposition in a community repository (e.g. GitHub). See the Nature Portfolio guidelines for submitting code & software for further information.

Data

Policy information about availability of data

All manuscripts must include a data availability statement. This statement should provide the following information, where applicable:

- Accession codes, unique identifiers, or web links for publicly available datasets
- A description of any restrictions on data availability
- For clinical datasets or third party data, please ensure that the statement adheres to our policy

Data supporting this work are available in this Article. The data generated in this study are provided in the Supplementary Information or source data file. The demo image data generated in this study, custom software script, MATLAB script and Fiji-java script are available in the GitHub database under accession code (https://github.com/zazadovv/Macrophage_Vessel.git; https://github.com/joeshen123/Nuclear-Membrane-Binding-Analysis.git). All other data supporting the findings of this study are available from corresponding author upon reasonable re-quest. Specifically, the time-lapse and multi-photon imaging data can be made available only upon request due to its proprietary file formats, data privacy laws, very large file sizes (Total>20TB) and permanent repository storage limitation. The primers are provided in the manuscript methods section, the plasmids and zebrafish strains created in this study are listed in methods section and are available with compliance in animal transfer agreements and institutional animal care IACUC approval.

Research involving human participants, their data, or biological material

Policy information about studies with human participants or human data. See also policy information about sex, gender (identity/presentation), and sexual orientation and race, ethnicity and racism.

Reporting on sex and gender	N/A
Reporting on race, ethnicity, or other socially relevant groupings	N/A
Population characteristics	N/A
Recruitment	N/A
Ethics oversight	N/A

Note that full information on the approval of the study protocol must also be provided in the manuscript.

Field-specific reporting

Please select the one below that is the best fit for your research. If you are not sure, read the appropriate sections before making your selection.

☒ Life sciences ☐ Behavioural & social sciences ☐ Ecological, evolutionary & environmental sciences

For a reference copy of the document with all sections, see nature.com/documents/nr-reporting-summary-flat.pdf

Life sciences study design

All studies must disclose on these points even when the disclosure is negative.

Sample size	No statistical methods were used to predetermine sample size. The chosen sample sizes are consistent with those reported in previous publications. In brief, experiments were performed on at least 8 independent animals to account for inter-sample variability, variance, and range, with maximal synchronization to osmotic and UV/FRAP wound responses. For IR-laser wounding and high-resolution imaging with the 63× objective, experiments were performed on at least 3 independent animals, each derived from separate clutches of embryos.
Data exclusions	The larvae were selected on the following criteria: normal morphology, a beating heart and circulating red blood cells. Data exclusion criteria were predefined. Animals were excluded if yolk and/or cardiac function did not restore after dextran injection ex-periments. The specimens were randomly chosen for pharmacological, chemogenetic genetic or transgenic imaging experiments without bias or pre-selection. Imaging

da-tasets were excluded if affected by artificial fluorescence fluctuations (e.g. dust debris), incompletely acquired Z-stacks or experiments where tailfin tissue drift exceeded 300 pixels (~150 microns) in the XY plane or 50 pixels (~25µm) in the Z-direction and were completely discarded, since no reliable data can be extracted. For x63 Oil objective im-aging, the live zebrafish at 2.5-4dpf do not have flat tail morphology and the depth of objective does not always reach the sample. Furthermore, Nikon's Perfect Focus Func-tion (PFS) was not able to focus an IR beam with the x63 objective, therefore most im-age data acquired drift upon shift in osmolyte or bathing medium, we have only re-tained data that do not show excessive XYZ drift in the imaging experiment. For each UV or IR-induced tissue wounding, directly damaged cells that were within the wound ROI ablation region were excluded from the analysis. No data was excluded based on distribution (no outlier exclusion). Details of statistical analyses used, exact P values, number of experiments/animals and sample sizes for all graphs are listed in figure or statistical source data file.

Replication	Animals used in the manuscript are 2.5-4 dpf old. The larvae derive from clutch of embryos that are collected from large breeding spawn with mixture of multiple adult zebrafish adults. Each experiment was replicated on at one or least two different calendar days and always included multiple biologically independent animals per calendar date. Animals were never reused across experiments, and all statistical analyses were performed across biological replicates. Replication incorporated variation in biological source, with experiments conducted on independent larvae derived from multiple parents per clutch across several or single experimental calendar date. For pharmacological treatments and vehicle controls, larvae were derived from the same spawn of embryos and experiments were repeated on at least two separate calendar dates. For transgenic larvae experiments, animals were derived from multiple independent clutches of embryos per calendar date. All attempts at replication were successful unless datasets met predefined exclusion criteria (e.g. excessive XY or Z-drift).
Randomization	The specimen in the imaging experiments were randomly allocated to experimental groups without any bias or pre-selection.
Blinding	Investigators were not blinded to allocation, except for genetic experiments with pla2g4aa, where all measurements were taken prior to knowledge of embryo genotype. Experimental blinding was not performed in other cases to maximize experimental throughput. Data analysis was performed blind to experimental condition, with image processing in Fiji and Napari completed prior to statistical testing in MATLAB or Prism.

Reporting for specific materials, systems and methods

We require information from authors about some types of materials, experimental systems and methods used in many studies. Here, indicate whether each material, system or method listed is relevant to your study. If you are not sure if a list item applies to your research, read the appropriate section before selecting a response.

Materials & experimental systems

n/a	Involved in the study
☒	☐ Antibodies
☒	☐ Eukaryotic cell lines
☒	☐ Palaeontology and archaeology
☐	☒ Animals and other organisms
☒	☐ Clinical data
☒	☐ Dual use research of concern
☒	☐ Plants

Methods

n/a	Involved in the study
☒	☐ ChIP-seq
☒	☐ Flow cytometry
☒	☐ MRI-based neuroimaging

Animals and other research organisms

Policy information about studies involving animals; ARRIVE guidelines recommended for reporting animal research, and Sex and Gender in Research

Laboratory animals	casper zebrafish; AB zebrafish. Adult casper and AB zebrafish were reared in 2.8-liter polycarbonate tanks at a density of ~8-10 fish per liter. Fish were maintained in salinity-conditioned system water at 28 °C under a 14:10-hour light:dark photoperiod cycle. All imaging experiments used in the manuscript are supported by larval zebrafish imaging experiments conducted at range of development 2.5-4 days post fertilization (dpf).. Only NTR2.0 experiments were conducted exclusively on 3 or 4dpf to allow 24 hrs for metronidazole or vehicle (DMSO) depletion before dextran perfusion and confocal imaging.
Wild animals	Study did not involve any wild animals.
Reporting on sex	Sex was indeterminate at 2.5–4 dpf and all experiments supporting findings of the manuscripts were conducted at these larval stages.
Field-collected samples	The study did not involve samples that were directly collected from the field.
Ethics oversight	Experiments were approved and conducted according to the institutional compliance and approval of the animal ethics committee, the Institutional Animal Care and Use Committee (IACUC) and the Research Animal Resource Center (RARC) of the Memorial Sloan Kettering Cancer Center (MSKCC, Protocol #11-01-002).

Note that full information on the approval of the study protocol must also be provided in the manuscript.

Plants

Seed stocks	N/A
Novel plant genotypes	N/A
Authentication	N/A
